# Prognostic Impact of Carboxylesterase 2 in Cholangiocarcinoma

**DOI:** 10.1038/s41598-019-40487-9

**Published:** 2019-03-13

**Authors:** Benjamin Goeppert, Marcus Renner, Stephan Singer, Thomas Albrecht, Qiangnu Zhang, Arianeb Mehrabi, Anita Pathil, Christoph Springfeld, Bruno Köhler, Christian Rupp, Karl Heinz Weiss, Anja A. Kühl, Ruza Arsenic, Ulrich Frank Pape, Arndt Vogel, Peter Schirmacher, Stephanie Roessler, Nalân Utku

**Affiliations:** 1 Institute of Pathology, University Hospital Heidelberg, Im Neuenheimer Feld 224, Heidelberg, Germany; 2 Liver Cancer Center Heidelberg (LCCH), University Hospital Heidelberg, Heidelberg, Germany; 3 Department of General Visceral and Transplantation Surgery, University Hospital Heidelberg, Im Neuenheimer Feld 110, Heidelberg, Germany; 4 Department of Internal Medicine IV, Gastroenterology and Hepatology, University Hospital Heidelberg, Im Neuenheimer Feld 410, Heidelberg, Germany; 5 University Hospital Heidelberg, National Center for Tumor Diseases, Department of Medical Oncology, Heidelberg, Germany; 6 Department of Gastroenterology-Immunpathology, Institute for Medical Immunology, Campus Steglitz, Berlin, Charité Germany; 7 Department of Pathology, Institute for Medical Immunology, Campus Mitte, Berlin, Charité Germany; 8 Asklepios Klinik St. Georg, Asklepios Kliniken Hamburg GmbH, Hamburg, Germany; 9 Department of Internal Medicine, Medizinische Hochschule Hannover, Hannover, Germany; 10 Institute for Medical Immunology, Campus Virchow, Berlin, Charité Germany; 11 CellAct Pharma GmbH, Otto Hahn Strasse 15, 44227 Dortmund, Germany

## Abstract

Carboxylesterase 2 (CES2) is instrumental for conversion of ester-containing prodrugs in cancer treatment. Novel treatment strategies are exceedingly needed for cholangiocarcinoma (CCA) patients. Here, we assessed CES2 expression by immunohistochemistry in a CCA cohort comprising 171 non-liver fluke associated, intrahepatic (n = 72) and extrahepatic (perihilar: n = 56; distal: n = 43) CCAs. Additionally, 80 samples of high-grade biliary intraepithelial neoplastic tissues and 158 corresponding samples of histological normal, non-neoplastic biliary tract tissues were included. CES2 expression was highest in non-neoplastic biliary tissue and significantly decreased in CCA. Patients showing any CES2 expression in tumor cells had a significantly better overall survival compared to negative cases (*p* = *0.008*). This survival benefit was also maintained after stratification of CES2-positive cases, by comparing low, medium and high CES2 expression levels (*p-trend* = *0.0006*). Evaluation of CCA subtypes showed the survival difference to be restricted to extrahepatic tumors. Correlation of CES2 expression with data of tumor-infiltrating immune cells showed that particularly CD8+ T cells were more frequently detected in CES2-positive CCAs. Furthermore, treatment of CCA cell lines with the prodrug Irinotecan reduced cell viability, increased cytotoxicity and modulated inflammatory gene expression. In conclusion, reduced CES2 expression is associated with poor outcome and low CD8+ T cell infiltration in CCA patients. Further clinical studies could show, whether CES2 expression may serve as a predictive marker in patients treated with prodrugs converted by CES2.

## Introduction

Cholangiocarcinomas (CCAs) are a diverse group of malignant epithelial tumors that may arise at any site of the biliary ducts. Three major clinical subtypes are defined: CCAs of intrahepatic (iCCA) or extrahepatic origin; the latter with perihilar (pCCA) or distal (dCCA) location. Clinically, CCA and even biliary tract cancers in general are often treated as one disease, although genetic heterogeneity and differences in clinical behavior are nowadays widely acknowledged^1^. Most CCA patients present with unresectable or metastatic disease. Despite systemic chemotherapy, prognosis remains poor and to date there are no established molecular targeted therapies tailored to biliary tract cancer^2^. Although the genomic spectra of CCA have been previously nicely depicted^3^, promising targeted therapy approaches are so far only available for a small subset of patients that are eligible to exploratory clinical trials. The treatment options and response for CCA remain still very discouraging as none of the treatment approaches yield an overall survival beyond one year^4^. Most of the treatment attempts including novel targeted therapies failed in trials so far. Thus, there is a high unmet need for the development of new drugs. Still, there are a number of open questions, not only concerning molecular alterations but also with respect to tumor-immune system interaction^5^. Detailed knowledge of this interaction could also be of interest in CCA patients, specifically in the era of combined conventional chemotherapeutics and tailored therapy approaches such as PD1/PD-L1 blockade^6^. The currently established first line standard treatment approach with gemcitabine and cisplatin cannot be regarded as an optimal therapeutic approach for all CCA subtypes as a variety of prognostic factors including metastatic disease, bilirubin, CEA & CA 19–9 levels, gender, ECOG, and RECIST criteria have been found to affect survival in this therapeutic regimen^7–10^. Interestingly, the effect of multiple factors is additive^2,11^. Thus, the overall effect of therapeutic regimens on survival may differ significantly in patient groups with different clinical presentation or biomarkers.

Carboxylesterases are members of the serine esterase superfamily and located in the endoplasmic reticulum and the cytosol of cells in many tissues, especially within the liver, the kidney, the small intestine and the colon^12,13^. Carboxylesterase 2 (CES2) is the predominant CES isoform in the gastrointestinal tract^14^. CES enzymes are instrumental to catalyze the hydrolysis of esters, amides, thioesters and carbamates, including environmental toxins and drugs, such as cytostatic drugs used in tumor treatment^15,16^. The enzymatic activity of CES2 can be exploited to cleave inactive prodrugs, releasing the active component of a prodrug at the site of a tumor^17^. Such examples are the hydrolysis of LY2334737 to gemcitabine, the conversion of irinotecan to 7-ethyl-10-hydroxy-camptothecin or the conversion of CAP7.1 to etoposide by CES2^4,18,19^. Interestingly, CES2 expression has been demonstrated to be deregulated by p53 and p38MAPK–NF-κB which are known to be involved in development and progression of multiple tumor entities^20,21^. Immunohistochemical evaluation has shown moderate expression of CES2 in normal human colon tissue and a broad range of expression levels in colorectal cancer with downregulation in the course of cancer progression^18,22,23^.

Regarding insight of CES2 expression in CCA patients, reliable data is missing. As CES-dependent prodrug approaches are currently utilized to treat CCA^24,25^, we aimed to analyze CES2 in a large and well-characterized CCA cohort, including all subtypes. Our findings may be of high clinical significance since CES2 expression was associated with patient outcome and may serve as biomarker for patient stratification.

## Materials and Methods

### Clinicopathological characteristics of cholangiocarcinoma patients

Tissue samples from 171 patients (median age 63.6 years) who underwent bile duct or liver surgery at the University Hospital Heidelberg between 1995 and 2010 were included in this study. This cohort was previously analyzed for tumoral immune cell infiltration^26^. The CCA cohort consisted only of adenocarcinomas, including all histologic variants. All CCA subtypes were represented adequately, including 72 intrahepatic CCAs and 99 extrahepatic CCAs (56 perihilar and 43 distal). Additionally, 80 samples of high-grade biliary intraepithelial neoplasia (BilIN 3) and 158 corresponding samples of histological non-neoplastic biliary tract tissues were included. Ampullary tumors were not included in this study, as they are often of intestinal histologic differentiation and represent a different tumor entity both clinically and biologically. None of the patients received radio- or chemotherapy prior to surgery. Tumors were restaged according to the 8^th^ TNM Classification of Malignant Tumors and classified after the World Health Organization (WHO) tumor classification system by two experienced pathologists^27^. A summary of clinicopathological data is given in Table 1 and Supplemental Tables 1 and 2. All experiments were performed in accordance with relevant guidelines and regulations. Informed consent for study participation was given by all patients according to the ethical guidelines of Heidelberg University Hospital. The use of the tissue specimens for this study was approved by the institutional ethics committee (S-207/2015).

**Table 1:** Clinicopathological data of the CCA cohort in correlation with absent (IRS = 0) compared to any (IRS = 1–12) CES2 expression score.

Number (percent)		total	CES2 score 0	CES2 score 1–12	p-value*
All CCA patients		171 (100.0)	54 (31.6)	117 (68.4)	0.870
Age	<*Median*^§^	86 (50.3)	28 (16.4)	58 (33.9)	
	>*Median*^§^	85 (49.7)	26 (15.2)	59 (34.5)	
Sex	*m*	112 (65.5)	31 (18.1)	81 (47.4)	0.166
	*w*	59 (34.5)	23 (13.5)	36 (21.1)	
CCA subgroups	*iCCA*	72 (42.1)	17 (9.9)	55 (32.2)	0.162
	*pCCA*	56 (32.7)	21 (12.3)	35 (20.5)	
	*dCCA*	43 (25.1)	16 (9.4)	27 (15.8)	
Histology	*ductal*	146 (85.4)	45 (26.3)	101 (59.1)	0.962
	*papillary*	11 (6.4)	4 (2.3)	7 (4.1)	
	*mucinous*	2 (1.2)	1 (0.6)	1 (0.6)	
	*intestinal*	6 (3.5)	2 (1.2)	4 (2.3)	
	*other*	6 (3.5)	2 (1.2)	4 (2.3)	
UICC#	*UICC 1*	11 (6.4)	3 (1.8)	8 (4.7)	0.568
	*UICC 2*	58 (33.9)	20 (11.7)	38 (22.2)	
	*UICC 3*	45 (26.3)	17 (9.9)	28 (16.4)	
	*UICC 4*	16 (9.4)	3 (1.8)	13 (7.6)	
	*NA*	41 (24.0)	11 (6.4)	30 (17.5)	
pT	*T1*	21 (12.3)	3 (1.8)	18 (10.5)	0.063
	*T2*	92 (53.8)	32 (18.7)	60 (35.1)	
	*T3*	43 (25.1)	11 (6.4)	32 (18.7)	
	*T4*	15 (8.8)	8 (4.7)	7 (4.1)	
pN	*N0*	55 (32.2)	17 (9.9)	38 (22.2)	0.848
	*N1*	71 (41.5)	24 (14.0)	47 (27.5)	
	*Nx*	45 (26.3)	13 (7.6)	32 (18.7)	
M	*M0*	155 (90.6)	51 (29.8)	104 (60.8)	0.397
	*M1*	16 (9.4)	3 (1.8)	13 (7.6)	
G	*G1*	8 (4.7)	2 (1.2)	6 (3.5)	0.339
	*G2*	121 (70.8)	35 (20.5)	86 (50.3)	
	*G3*	42 (24.6)	17 (9.9)	25 (14.6)	
L	*L0*	88 (51.5)	27 (15.8)	61 (35.7)	0.870
	*L1*	83 (48.5)	27 (15.8)	56 (32.7)	
V	*V0*	125 (73.1)	43 (25.1)	82 (48.0)	0.265
	*V1*	46 (26.9)	11 (6.4)	35 (20.5)	
R	*R0*	79 (46.2)	23 (13.5)	56 (32.7)	
	*R1*	54 (31.6)	18 (10.5)	36 (21.1)	0.927
	*R2*	13 (7.6)	4 (2.3)	9 (5.3)	
	*Rx*	25 (14.6)	9 (5.3)	16 (9.4)	
Pn	*Pn0*	99 (57.9)	26 (15.2)	73 (42.7)	0.096
	*Pn1*	72 (42.1)	28 (16.4)	44 (25.7)	

^§^Median age: 63.6 years.

*Fisher’s exact test; not available data (NA).

^#^Cases with pNx had no lymph nodes resected, therefore, UICC status could not be assessed.

### Tissue microarray construction

From all 171 CCA FFPE tissue blocks, 3 µm sections were cut and stained with H&E. Representative areas were marked by two experienced pathologists (BG and SS). For each case, tumor tissue cores (1.0 mm diameter) from the selected representative tumor areas were punched out of the sample tissue blocks and embedded into a new paraffin array block using a tissue microarrayer (Beecher Instruments, Woodland, CA, USA).

### Immunohistochemistry and tissue microarray evaluation

**Figure 1:**
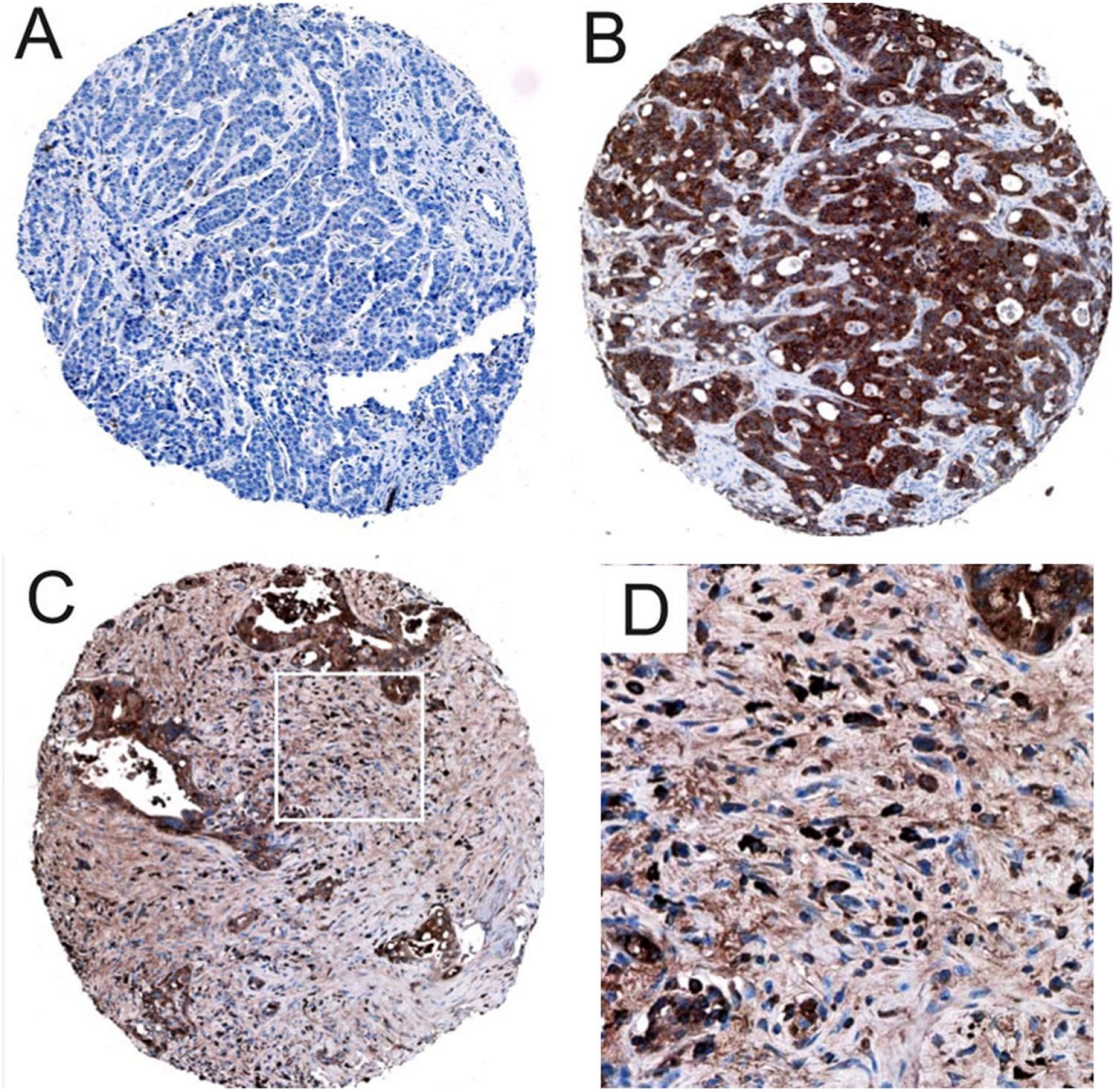
CES2 immunohistochemistry of representative CCA cases. Representative TMA core of an iCCA, negative for CES2 by immunohistochemistry (**A**). TMA core of an iCCA showing homogenous strong cytoplasmic CES2 immunoreactivity, while tumor stroma is CES2 negative (**B**). TMA core of a pCCA showing homogenous strong cytoplasmic CES2 immunoreactivity (**C**), while tumor stromal cells are also partly CES2 immunoreactive (**D**). Original magnification: 40x (**A**–**C**), 200x (**D**).

Immunohistochemical analyses were performed on 3 μm thick Tissue Microarrays (TMAs). As previously described for the analysis of CES2 immunohistochemistry, sections of archived patient samples were subjected to heat-induced epitope retrieval prior to incubation with a rabbit antibody specific for CES2 (ab111751, Abcam; Cambridge, United Kingdom)^12^. CES2 immunoreactivity was visualized using the EnVision + HRP System (K4011; Dako, Glostrup, Denmark) directed against rabbit primary antibodies with diaminobenzidine (DAB; Dako) as chromogen. The nuclei were counterstained with hematoxylin and slides cover slipped with glycerol gelatin (both Merck, Darmstadt, Germany). CES2 immunoreactivity was seen in CCA tumor cells and in the tumor stroma component (Fig. 1). Both components were evaluated separately. For the epithelial tumor cell component, a semi-quantitative immunohistochemical assessment of cytoplasmic CES2 expression, the product of the scores of staining intensity and percentage of immunoreactive cells was calculated based on the following scoring system: the intensity ranged from 0 = negative, 1 = low, 2 = medium to 3 = high; the quantity comprised 0 = no expression, 1 = positivity in less than 10%, 2 = positivity in 10% to 50%, 3 = positivity in 51% to 80%, and 4 = positivity in more than 80% of biliary cells (non-neoplastic tissue/BilIN 3/CCA). The final immunoreactive score was obtained by multiplication of the intensity score and the quantity score according to immunoreactive score (IRS, ranging from 0 to 12). For comparison of staining results, we further defined a scoring index comprising three different expression scores for CES2 based on the calculated product of cytoplasmic intensity and quantity of immunoreactive cells: 0–4 = absent/low expression; 5–8 = moderate expression; 9–12 = high expression. For stromal CES2 expression, cytoplasmic staining in stromal cells was evaluated separately and was assessed in a four-tiered manner (0 = no expression; 1 = low expression; 2 = moderate expression; 3 = high expression). Evaluation was performed independently by two experienced pathologists (BG, SS).

### Cell line culture, treatment, cell viability and cytotoxicity assays

EGI-1 cells were cultured in Dulbecco’s Modified Eagle’s Medium (DMEM) and TFK¬1 were maintained in RPMI-1640 medium supplemented with 10% fetal calf serum and 1% Penicillin-streptomycin (100 IU/mL and 100 g/mL, respectively). All media and supplements were obtained from Sigma-Aldrich (St. Louis, MO, USA). Cells were incubated at 37 °C with 5% CO2. Cells were incubated with 20 µg/ml Irinotecan or left untreated.

For the cell viability and cytotoxicity assays, 5,000 TFK-1 or EGI-1 cells per well were seeded into a 96-well plate in quadruplicates. Cells were incubated with 20 μg/ml Irinotecan or left untreated for up to 3 days and measurements were performed at 0, 48 and 72 h. For cytotoxicity measurement, CellTox™ Green Cytotoxicity Assay (Promega, Madison, WI, USA) was used. CellTox™ Green was 1:1000-diluted with assay buffer, added to the cells and incubated for 15 min at RT in the dark and measured (485 nm Ex/520 nm Em) with an Omega FLUOstar Microplate Reader (BMG LABTECH, Ortenberg, Germany). For cell viability measurement, growth medium containing 10% Resazurin (R&D Systems, Minneapolis, MN, USA) was added to the cells, incubated for 1 h at 37 °C and measured (544 nm Ex/590 nm Em) with an Omega FLUOstar Microplate Reader (BMG LABTECH). All experiments were repeated three times in quadruplicates for both cell lines.

### RNA extraction, cDNA synthesis and semi-quantitative reverse-transcription polymerase chain reaction (qRT-PCR)

For the qRT-PCR assay, cells were seeded into 6-well plates treated with 20 μg/ml Irinotecan or left untreated for 48 h. Total RNA was extracted with ExtractMe Total RNA Kit (Blirt, Gdansk, Poland) according to the manufacturer’s protocol. cDNA was synthesized from 1 μg total RNA using RevertAid H Minus First Strand cDNA Synthesis Kit (Thermo Fisher Scientific, Offenbach, Germany). Samples of three independent experiments were analyzed in duplicates using primaQUANT CYBR Master Mix (Steinbrenner Laborsysteme, Wiesenbach, Germany) on a StepOnePlus real-time PCR instrument (Applied Biosystems, Darmstadt, Germany). The human reference gene serine/arginine-rich splicing factor 4 (SRSF4) was used as an internal control. Relative mRNA expression values were calculated using the comparative Ct method. Primers were obtained from Thermo Fisher Scientific and had the following sequence: 1. SRSF4 (NM_005626.4) Fwd: 5′-TGCAGCTGGCAAGACCTAAA-3′ and Rev: 5′-TTTTTGCGTCCCTTGTGAGC-3′; 2. STAT1 (NM_007315.3) Fwd: 5′-ACCTAACGTGCTGTGCGTAG-3′ and Rev: 5′-GGTGAACCTGCTCCAGGAAT-3′; 3. MX1 (NM_001144925.2) Fwd: 5′-TGGCATAACCAGAGTGGCTG-3′ and Rev: 5′-CCACATTACTGGGGACCACC-3′; 4. PARP9 (NM_001146106.1) Fwd: 5′-GGCCACATTGAATGGCAGAC-3′ and Rev: 5′-TACCAACTGGGACCGTTGAA-3′, 5. IFI27 (NM_001130080.2) Fwd: 5′-GGAATTAACCCGAGCAGGCA-3′ and Rev: 5′-ATGGCCACAACTCCTCCAATC-3′; 6. IL8 (NM_000584.4) Fwd: 5′-GAGTGGACCACACTGCGCCA-3′ and Rev: 5′-TCCACAACCCTCTGCACCCAGT-3′; TNF (NM_000594.4) Fwd: 5′- ACTTTGGAGTGATCGGCCC-3′ and Rev: 5′- CATTGGCCAGGAGGGCATT-3′.

### Statistical analyses including correlation analyses with other immunohistochemical variables

Statistical analyses were performed with the statistical computing environment R version 3.0.1. Missing values were omitted from the analysis. Correlation analyses of CES2 expression status with clinicopathological and other immunohistochemical variables were assessed with Fisher’s exact test. Univariate survival analysis was performed for overall survival by generation of Kaplan-Meier curves. Significance of differences between the groups was assessed using the Log-rank-test. P values < 0.05 were considered significant. Data concerning expression of PD-L1, MHC I, quantity and quality of tumor infiltrating immune cells, as well as proliferation index (Ki-67) were available partly from previously published studies^26,28^.

## Results

### CES2 expression in cholangiocarcinoma including all subtypes

**Figure 2:**
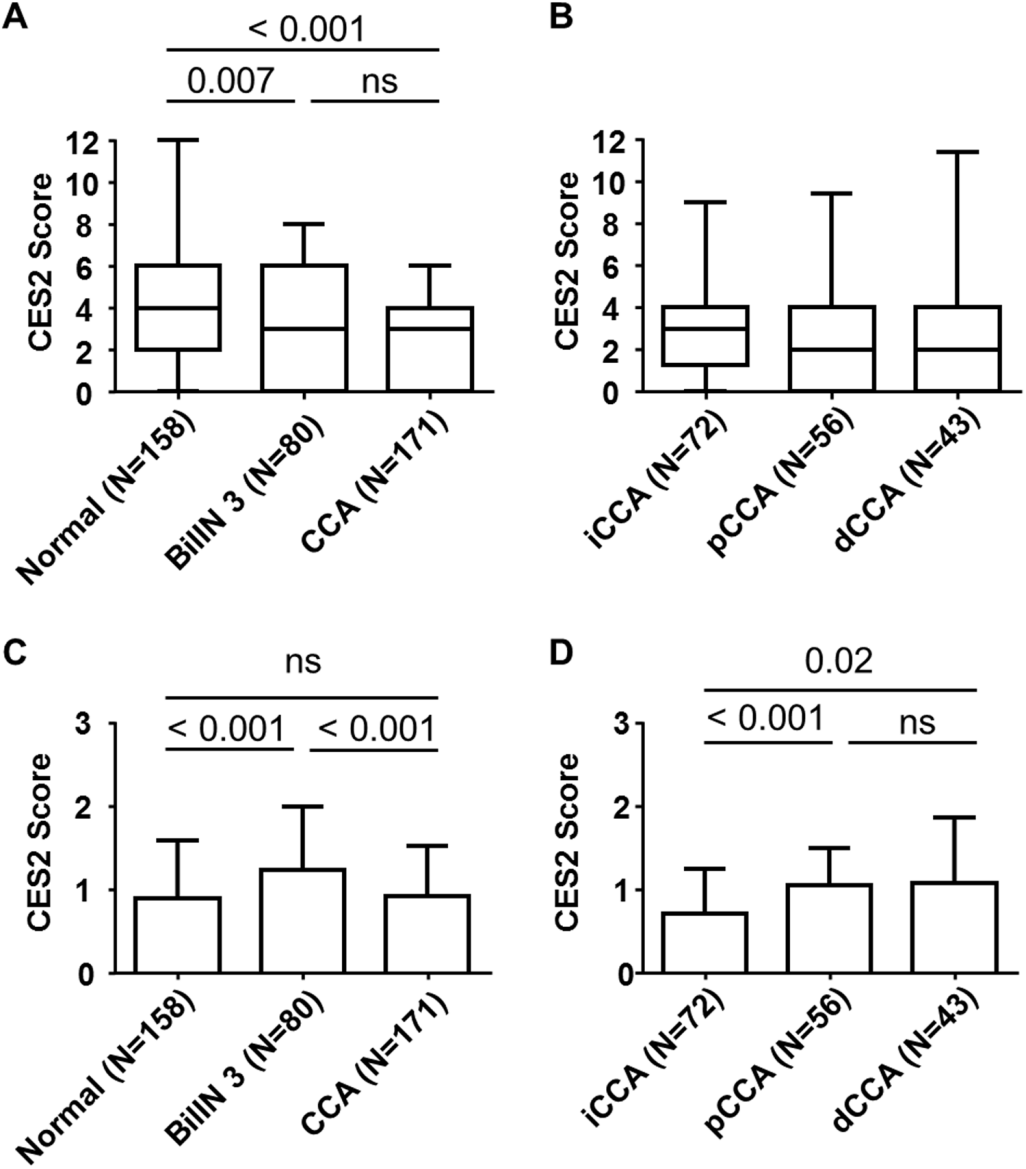
CES2 immunoreactivity in non-neoplastic normal biliary epithelium, high-grade biliary intraepithelial neoplasias and invasive CCA including subtype-specific evaluation. CES2 immunoreactivity was highest in non-neoplastic, normal biliary epithelium and stepwise decreasing in cholangiocarcinogenesis (**A**; *p* < 0.001, *p* = 0.004 respectively). No significant differences in CES2 immunoreactivity were detected in CCA subtypes (**B**). P-values were calculated by Mann-Whitney U test. Box plot with 5–95% whiskers.

A distinct CES2 immunoreactivity was widely detectable in CCA tissues. CES2 immunohistochemistry showed a distinct cytoplasmic staining signal (Fig. 1). CES2 immunoreactivity was evaluated separately in CCA tumor cells and in the tumoral stroma component (see Material & Methods section). CES2 immunoreactivity decreased significantly from non-neoplastic “normal” biliary epithelium to high-grade dysplasia (BilIN grade 3) to invasive CCA, but did not differ significantly between CCA subtypes (*p* = 0.007, *p* < 0.001 respectively; Fig. 2A,B). Stromal evaluation showed significantly higher CES2 immunoreactivity in high grade dysplasia (BilIN grade 3) compared to non-neoplastic “normal” biliary epithelium and invasive CCA (*p* < 0.001 for both analyses, Fig. 2C). In addition, CES2 immunoreactivity in tumoral stroma of CCA was increased in pCCA and dCCA compared to iCCA (*p* < 0.001 and *p* = 0.02, respectively; Fig. 2D).

### Correlation of CES2 immunoreactivity with overall survival and clinicopathological data of cholangiocarcinoma patients

**Figure 3:**
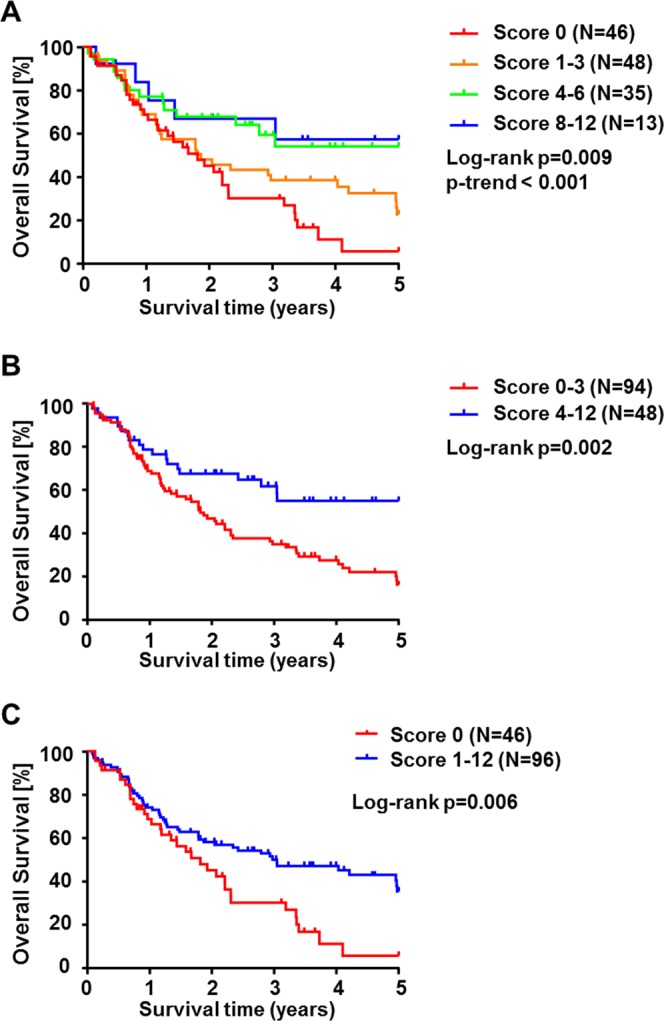
Overall survival probability in cholangiocarcinoma patients in correlation with CES2 immunoreactivity. Kaplan-Meier curves show a longer overall survival of CCA patients in correlation with higher CES2 immunoreactive scores (**A**). CCA patients with low (**B**) or absent (**C**) CES2 immunoreactivity also showed a significant shortened overall survival compared to patients with higher CES2 immunoreactive scores. *P*-values were calculated by log-rank test or p-trend (**A**). Survival data were available for 142 (83%) of 171 CCA patients.

**Figure 4:**
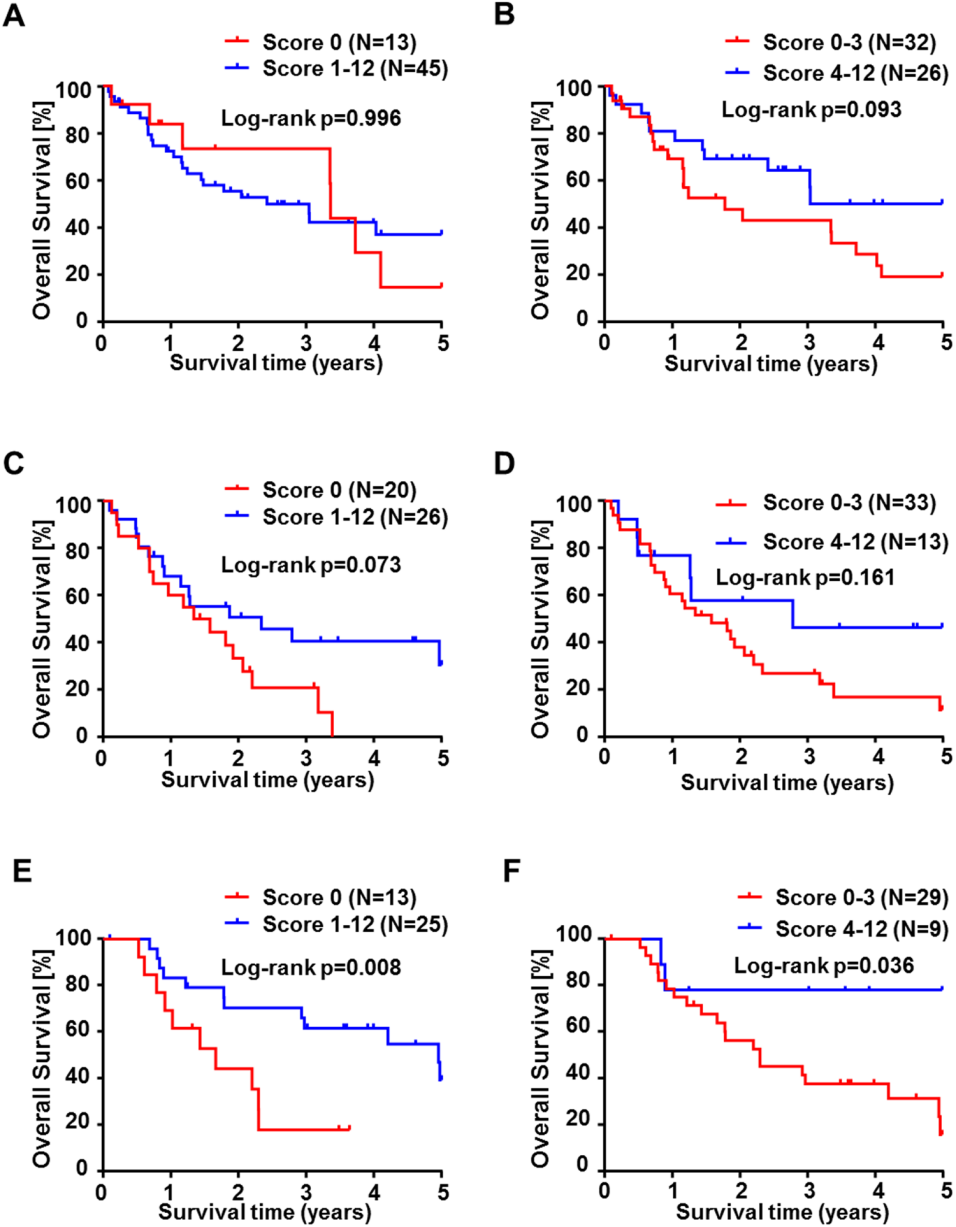
Overall survival probability in cholangiocarcinoma subtypes in correlation with CES2 immunoreactivity. Kaplan-Meier curves show no significant overall survival differences of iCCA (**A**,**B**) and pCCA (**C**,**D**) patients in correlation with CES2 immunoreactivity, whereas a significant longer overall survival of dCCA patients in correlation with CES2 immunoreactivity was detected (**E**, *p* = 0.008; **F**, *p* = 0.036). *P*-values were calculated by log-rank test.

**Figure 5:**
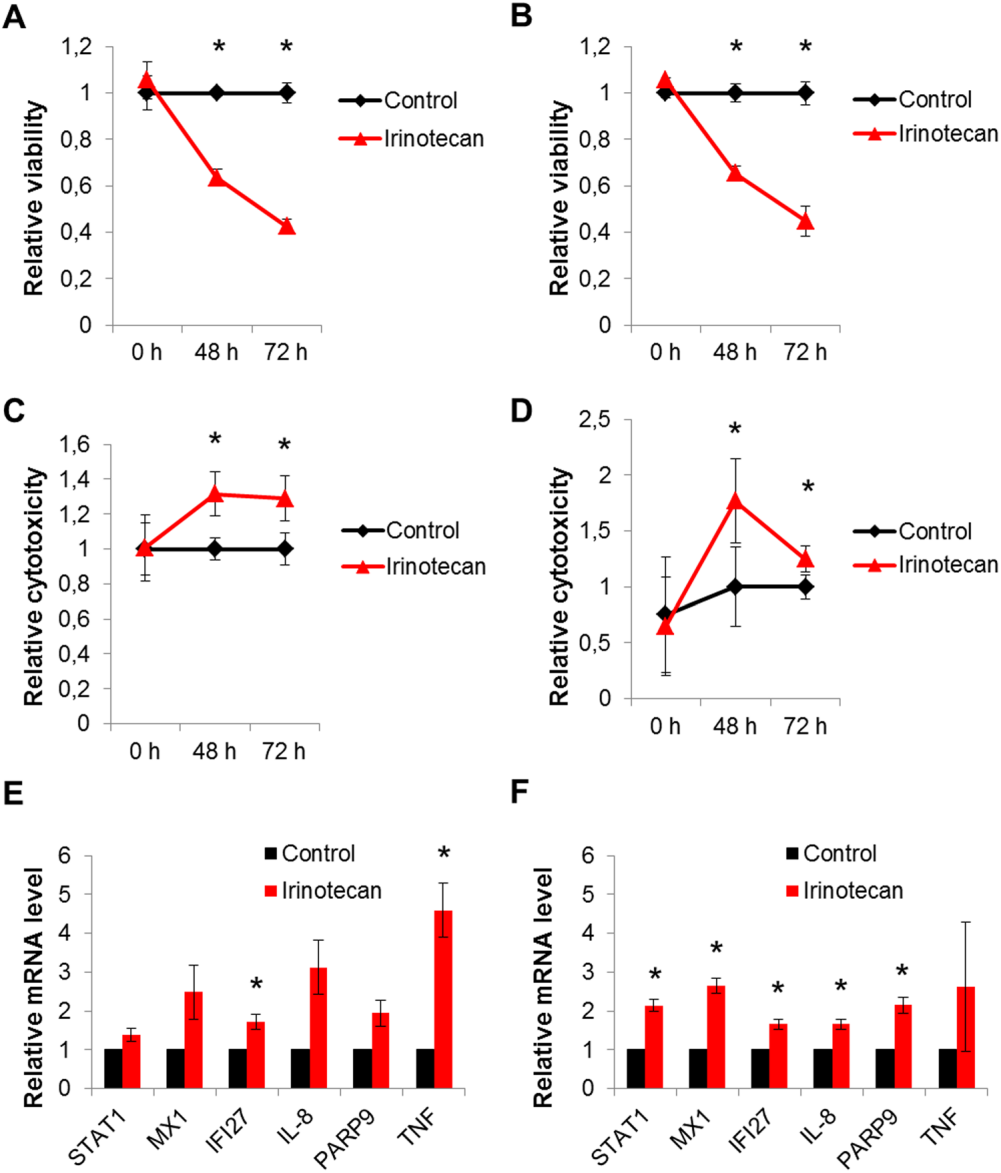
The prodrug Irinotecan reduces cell viability, induces cytotoxicity, and induces the expression of inflammatory genes in CCA cell lines. Cell viability assay of TFK-1 (**A**) and EGI-1 (**B**) and cytotoxicity assays of TFK-1 (**C**) and EGI-1 (**D**) upon treatment with 20 μg/ml Irinotecan for up to 72 h. Control cells were untreated. Shown are relative mean values with standard deviation (SD) of one out of three independent experiments with similar results. Within each experiment values were normalized to control treated cells. Relative mRNA levels of TFK-1 and EGI-1 cells treated with 20 μg/ml Irinotecan or left untreated for up to 72 h **(E**,**F**). Shown are relative mean values with SD of three independent experiments. Within each experiment values were normalized to control treated cells. *Mann-Whitney U test p < 0.05.

Correlation analysis of CES2 immunoreactivity score in CCA showed a significantly shortened overall survival in patients with lower CES2 scores (*p* = 0.009; *p-trend* < 0.001; Fig. 3A). We first compared different groups of CES2 expression and found that CCA patient groups with low (IRS = 0–3) or none (IRS = 0) tumoral CES2 immunoreactivity both had significantly shortened overall survival (*p* = 0.002, Fig. 3B; *p* = 0.006, Fig. 3C). Subgroup analysis revealed that this finding is due to differential expression in extrahepatic CCAs, mainly dCCA, which consisted of significantly less CES2 negative or low expressor cases (Fig. 4). In contrast, in iCCA no significant correlation between CES2 immunoreactivity and patient survival could be detected (Fig. 4A,B). Correlation analyses of CES2 immunoreactivity in tumor stroma did not correlate significantly with overall survival rates of CCA patients, even when calculated subtype-specifically (Suppl. Figs 1, 2). In addition, analysis of comprehensive clinicopathological data of this CCA cohort showed no consistent differences between patients with low or high CES2 expression (Table 1 and Suppl. Table 1). Furthermore, we functionally tested *in vitro* the effect of the prodrug Irinotecan which is mainly converted by CES2 in two CCA cell lines, TFK-1 and EGI-1. Treatment of TFK-1 and EGI-1 with 20 μg/ml Irinotecan led to significantly reduced cell viability and increased cytotoxicity in both cell lines (Fig. 5A–D). Furthermore, Irinotecan treatment induced mRNA expression of inflammation-related genes, such as STAT1, MX1, IFI27, IL-8, PARP9 and TNF (Fig. 5E,F). This demonstrated that Irinotecan may be functionally active in CES2-positive CCA.

### CES2 expression and tumor infiltrating immune cells in cholangiocarcinoma

**Figure 6:**
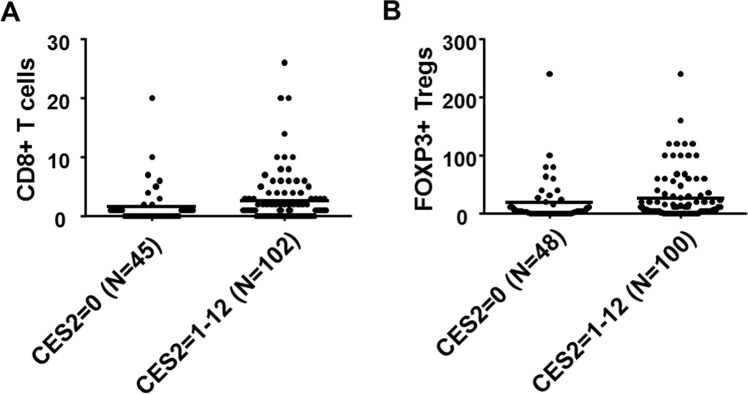
Immune cell infiltration in CES2-negative and -positive CCA. Analysis of quantity of CD8+ intraepithelial T cells in correlation with CES2 expression in the CCA cohort (**A**; Mann Whitney test *p* = 0.046) and analysis of quantity of total FOXP3+ regulatory T cells in the CCA cohort (**B**; Mann Whitney test *p* = 0.046).

Since it is known that tumor infiltrating immune cells are of prognostic value in CCA, we performed a correlation analysis of CES2 immunoreactive score with previously published data on the quantity and quality of tumor infiltrating immune cells^26^. This revealed an increase in the presence of inflammatory cells, particularly intratumoral CD8+ T cells and tumor infiltrating FOXP3+ regulatory T cells in CES2-positive CCAs (*p* = 0.046 each; Fig. 6). Other immune cell types (CD4+/FOXP3-, CD20+, and CD68+) were not increased in association with increased CES2 staining (Suppl. Fig. 3). Stromal CES2 expression was also not associated with different immune cell infiltrates (Suppl. Fig. 4). Since MHC class I expression on CCA tumor cells has also been described as a prognostic factor^28^, we correlated MHC class I antigen expression with CES2 immunoreactivity. However, no significant association was found in our CCA cohort (Suppl. Fig. 5). Thus, patients exhibiting high CES2 expression show a distinct tumor immune profile with increased presence of CD8+ and FOXP3+ immune cells.

## Discussion

CES2 expression differs in non-neoplastic tissues and varies between different tumor types including colorectal cancer (CRC), pancreatic ductal adenocarcinoma (PDAC), lung adenocarcinoma and breast cancer^12,18,29^. Furthermore, high CES2 expression in tumor tissue was associated with longer overall survival in resectable and borderline resectable PDAC patients who underwent neoadjuvant FOLFIRINOX treatment^29^. Thus, the assessment of CES2 expression for patient stratification prior to treatment with prodrugs converted mainly via CES2 might contribute significantly to clinical outcome.

Here, we evaluated CES2 expression by immunohistochemistry in tumor cells and tumoral stroma in a well-characterized and comprehensive cohort of non-liver fluke associated CCA using a CES2 antibody. Using the well-known immunoreactive score (IRS), a clear-cut read-out of CES2 expression could be performed. Since CES2 expression might be of therapeutic relevance in the future, we sought to establish a simple method and read-out to test CCA samples for CES2 expression to be used in clinical settings.

CES2 expression levels decreased significantly with the progression of cholangiocarcinogenesis, i.e. highest CES2 immunoreactive scores were seen in non-neoplastic normal biliary epithelium, while high-grade biliary intraepithelial neoplasias (BilIN grade 3) and invasive CCA showed a stepwise decrease of CES2 immunoreactivity. Interestingly, CES2 immunoreactivity in tumoral stroma was highest in BilIN grade 3, whereas, non-neoplastic normal biliary epithelium and CCA did not differ significantly with regard to stromal CES2 immunoreactivity. CES2 immunoreactivity in CCA did not show any differences in the subtype-specific evaluation but in the tumoral stroma highest CES2 expression scores were detected in extrahepatic pCCA and dCCA, while iCCA showed significant lower CES2 immunoreactivity in the tumoral stroma. High CES2 expression was associated with longer overall survival of CCA patients. Conversely, this finding was corroborated by subgrouping patients into low or non-CES2 expressors, both of which exhibited a significantly lower overall patient survival (Fig. 3B,C). Thereby, a simple and prognostically relevant testing method for CES2 expression in CCA patients was established. Consequently, we tested this survival association in CCA subtypes, thereby revealing that this effect is mostly due to extrahepatic, in specific dCCA, since iCCA did not show any survival differences with respect to CES2 expression levels. These findings strengthen the current concepts of CCA subtypes, in particular the nowadays acknowledged molecular and also clinical differences between extrahepatic and intrahepatic CCA. Combining the findings of higher CES2 expression scores in tumoral stroma of pCCA and dCCA and the subtype-specific survival benefit of higher CES2 expression in dCCA and in pCCA, one is tempted to speculate on the impact of CES2 expression in tumoral stroma on the tumor – immune system interaction in extrahepatic CCA, although stromal CES2-expression levels were not significantly associated with survival in CCA and subtypes.

A study using an intracranial human glioma xenograft mouse model suggested that CES activity may induce higher peripheral CD4+ und CD8+ T cell degranulation which is a measure of potential immune response^30^. Furthermore, tumor-infiltrating immune cells have been suggested to be a source of CES2 and may be involved in the therapeutic efficacy of prodrugs^12,31^. High FoxP3+ regulatory T cell (Treg) infiltration is significantly associated with shorter survival in cervical, renal, breast cancers and malignant melanomas, whereas, high FoxP3+ Tregs are associated with improved survival in colorectal, head and neck, and oesophageal cancers^32^.

In our CCA cohort, we previously found that high numbers of intraepithelial CD8+ T cells, FOXP3+ Tregs, and high/moderate MHC I expression levels are correlated with better patient survival^26,28^. In the present study, we observed higher intratumoral CD8+ T cells and FOXP3+ T cells in CES2-positive CCAs (Fig. 6). Therefore, an impact of CES2 on the tumor - immune system interaction is conceivable.

Consistent with previous data obtained in other tumor entities, we showed that Irinotecan treatment inhibited cell viability, whereas it induced cytotoxicity and inflammation-related gene expression^33,34^. However, further functional studies are required to determine immune cell specificity and function with regard to different CES2-expression levels. Limitations of this study include the retrospective character, the focus on immunohistochemical analyses, and the missing animal studies which will be addressed in future studies.

Conclusively, we present a feasible method of evaluating CES2 expression, a novel prognostic marker in CCA. Our findings show subtype-specificity in CCA and indicate an impact of CES2 in the tumor – immune system interaction and direct anti-proliferative effect of CES2 conversion-dependent prodrug in CCA cell lines. The presented findings are likely to gain clinical relevance, as several prodrugs utilized in cancer are either in development or already available.

## Supplementary information

Supplementary Material

## References

[1] Banales JM (2016). Expert consensus document: Cholangiocarcinoma: current knowledge and future perspectives consensus statement from the European Network for the Study of Cholangiocarcinoma (ENS-CCA). Nature reviews. Gastroenterology & hepatology.

[2] Agarwal R (2016). Advanced biliary tract cancer: clinical outcomes with ABC-02 regimen and analysis of prognostic factors in a tertiary care center in the United States. J Gastrointest Oncol.

[3] Nakamura H (2015). Genomic spectra of biliary tract cancer. Nature genetics.

[4] Keilholz U (2017). First-in-man dose escalation and pharmacokinetic study of CAP7.1, a novel prodrug of etoposide, in adults with refractory solid tumours. Eur J Cancer.

[5] Abou-Alfa GK (2016). Advances in cholangiocarcinoma research: report from the third Cholangiocarcinoma Foundation Annual Conference. J Gastrointest Oncol.

[6] Sabbatino F (2016). PD-L1 and HLA Class I Antigen Expression and Clinical Course of the Disease in Intrahepatic Cholangiocarcinoma. Clin Cancer Res.

[7] Okusaka T (2010). Gemcitabine alone or in combination with cisplatin in patients with biliary tract cancer: a comparative multicentre study in Japan. Br J Cancer.

[8] Lamarca A, Benafif S, Ross P, Bridgewater J, Valle JW (2015). Cisplatin and gemcitabine in patients with advanced biliary tract cancer (ABC) and persistent jaundice despite optimal stenting: Effective intervention in patients with luminal disease. Eur J Cancer.

[9] Ji JH (2015). Natural history of metastatic biliary tract cancer (BTC) patients with good performance status (PS) who were treated with only best supportive care (BSC). Jpn J Clin Oncol.

[10] Cho H (2017). Prognostic factors in patients (pts) with advanced biliary tract cancer (BTC) treated with first-line gemcitabine plus cisplatin (GEMCIS): Retrospective analysis of 740 pts. Journal of Clinical Oncology.

[11] Komaya K (2017). Recurrence after resection with curative intent for distal cholangiocarcinoma. Br J Surg.

[12] Kuhl AA (2016). Tissue-infiltrating plasma cells are an important source of carboxylesterase 2 contributing to the therapeutic efficacy of prodrugs. Cancer Lett.

[13] Satoh T, Hosokawa M (2006). Structure, function and regulation of carboxylesterases. Chem Biol Interact.

[14] Taketani M, Shii M, Ohura K, Ninomiya S, Imai T (2007). Carboxylesterase in the liver and small intestine of experimental animals and human. Life Sci.

[15] Imai T, Taketani M, Shii M, Hosokawa M, Chiba K (2006). Substrate specificity of carboxylesterase isozymes and their contribution to hydrolase activity in human liver and small intestine. Drug Metab Dispos.

[16] Laizure SC, Herring V, Hu Z, Witbrodt K, Parker RB (2013). The role of human carboxylesterases in drug metabolism: have we overlooked their importance?. Pharmacotherapy.

[17] Tsuji T (1991). CPT-11 converting enzyme from rat serum: purification and some properties. J Pharmacobiodyn.

[18] Xu G, Zhang W, Ma MK, McLeod HL (2002). Human carboxylesterase 2 is commonly expressed in tumor tissue and is correlated with activation of irinotecan. Clin Cancer Res.

[19] Pratt SE (2013). Human carboxylesterase-2 hydrolyzes the prodrug of gemcitabine (LY2334737) and confers prodrug sensitivity to cancer cells. Clin Cancer Res.

[20] Mao Z (2011). Lipopolysaccharide down-regulates carbolesterases 1 and 2 and reduces hydrolysis activity *in vitro* and *in vivo* via p38MAPK-NF-kappaB pathway. Toxicol Lett.

[21] Xiao D (2013). Regulation of carboxylesterase-2 expression by p53 family proteins and enhanced anti-cancer activities among 5-fluorouracil, irinotecan and doxazolidine prodrug. Br J Pharmacol.

[22] Tang X (2008). Carboxylesterase 2 is downregulated in colorectal cancer following progression of the disease. Cancer Invest.

[23] Zhang C (2014). *In vitro* evaluation of the inhibitory potential of pharmaceutical excipients on human carboxylesterase 1 A and 2. PLoS One.

[24] Endlicher E (2016). Irinotecan Plus Gemcitabine and Fluorouracil in Advanced Biliary Tract Cancer: A Retrospective Study. Digestion.

[25] Konstantinidis IT (2016). Unresectable intrahepatic cholangiocarcinoma: Systemic plus hepatic arterial infusion chemotherapy is associated with longer survival in comparison with systemic chemotherapy alone. Cancer.

[26] Goeppert B (2013). Prognostic impact of tumour-infiltrating immune cells on biliary tract cancer. Br J Cancer.

[27] Bosman, F. T., Carneiro, F., Hruban, R. H. & Theise, N. D. *WHO Classification of Tumours of the Digestive System*. 4th edn, (International Agency for Research on Cancer (IARC), 2010).

[28] Goeppert B (2015). Major histocompatibility complex class I expression impacts on patient survival and type and density of immune cells in biliary tract cancer. Br J Cancer.

[29] Capello, M. *et al*. Carboxylesterase 2 as a Determinant of Response to Irinotecan and Neoadjuvant FOLFIRINOX Therapy in Pancreatic Ductal Adenocarcinoma. *J Natl Cancer Inst* **107**, 10.1093/jnci/djv132 (2015). 10.1093/jnci/djv132 PMC4554193 26025324

[30] Metz MZ (2013). Neural stem cell-mediated delivery of irinotecan-activating carboxylesterases to glioma: implications for clinical use. Stem Cells Transl Med.

[31] Cecchin E (2005). Carboxylesterase isoform 2 mRNA expression in peripheral blood mononuclear cells is a predictive marker of the irinotecan to SN38 activation step in colorectal cancer patients. Clin Cancer Res.

[32] Shang B, Liu Y, Jiang SJ, Liu Y (2015). Prognostic value of tumor-infiltrating FoxP3+ regulatory T cells in cancers: a systematic review and meta-analysis. Sci Rep.

[33] Iwata H (2016). PARP9 and PARP14 cross-regulate macrophage activation via STAT1 ADP-ribosylation. Nat Commun.

[34] Wang K (2014). A meta-analysis approach for characterizing pan-cancer mechanisms of drug sensitivity in cell lines. PLoS One.

