# Supplementary material for: Prognostic Impact of Carboxylesterase 2 in Cholangiocarcinoma
Source: Sci Rep. 2019 Mar 13;9:4338. doi: 10.1038/s41598-019-40487-9 (PMC6416336; doi:10.1038/s41598-019-40487-9)
Supplement: Supplementary file 1 — Supplementary Material [file 41598_2019_40487_MOESM1_ESM.docx]

***Supplemental material***

**Prognostic Impact of Carboxylesterase 2 in Cholangiocarcinoma**

Benjamin Goeppert^1,2*^, Marcus Renner^1^, Stephan Singer^1,2^, Thomas Albrecht^1,2^, Qiangnu Zhang^1^, Arianeb Mehrabi^3,2^, Anita Pathil^4^, Christoph Springfeld^5,2^, Bruno Köhler^5,2^, Christian Rupp^4,2^, Karl Heinz Weiss^4,2^, Anja A. Kühl^7^, Ruza Arsenic ^8^, Ulrich Frank Pape^9^, Arndt Vogel^6^, Peter Schirmacher^1,2^, Stephanie Roessler^1,2#^, and Nalân Utku^10,11*#^

^1^Institute of Pathology, University Hospital Heidelberg, Im Neuenheimer Feld 224, Heidelberg, Germany

^2^Liver Cancer Center Heidelberg (LCCH)

^3^Department of General Visceral and Transplantation Surgery, University Hospital Heidelberg, Im Neuenheimer Feld 110,

Heidelberg, Germany

^4^Department of Internal Medicine IV, Gastroenterology and Hepatology, University Hospital Heidelberg, Im Neuenheimer Feld 410, Germany

^5^University Hospital Heidelberg, National Center for Tumor Diseases, Department of Medical Oncology

^6^Department of Internal Medicine, Medizinische Hochschule Hannover, Germany

^7^Department of Gastroenterology - Immunpathology, Campus Steglitz; Department of Gastroenterology, Campus Mitte^9^ and ^8^Pathology, Campus Mitte and ^10^Institute for Medical Immunology, Campus Virchow, Berlin, Charité, Germany

^11^CellAct Pharma GmbH, Otto Hahn Strasse 15, 44227 Dortmund, Germany

^#^ Stephanie Roessler and Nalân Utku contributed equally to this work and therefore share last authorship

^*^*Corresponding authors:*

Benjamin Goeppert, Institute of Pathology, University Hospital Heidelberg,

Im Neuenheimer Feld 224, 69120 Heidelberg, Germany

Email: [benjamin.goeppert@med.uni-heidelberg.de](mailto:benjamin.goeppert@med.uni-heidelberg.de)

Nalân Utku, Institute for Medical Immunology, Campus Virchow,

Augustenburger Platz 1, 13353 Berlin, Universitätshospital Charité, Germany

Email: [nalan.utku@charite.de](mailto:nalan.utku@charite.de)

**Tables:**

**Supplemental Table 1:** *Clinicopathological data of cholangiocarcinoma cohort and correlation to CES2 immunoreactivity.*

Clinicopathological data of the CCA cohort in correlation with absent or low (IRS=0-3) compared to moderate to high (IRS=4-12) CES2 expression score.

| **Number (percent)** |  | **total** | **CES2 score 0-3** | **CES2 score 4-12** | **p-value** |
| --- | --- | --- | --- | --- | --- |
| **All CCA patients** |  | 171 (100.0) | 114 (66.7) | 57 (33.3) |  |
| **Age** | *< Median^§^* | 86 (50.3) | 62 (36.3) | 24 (14.0) |  |
|  | *> Median* | 85 (49.7) | 52 (30.4) | 33 (19.3) | 0.146 * |
| **Sex** | *m* | 112 (65.5) | 68 (39.8) | 44 (25.7) |  |
|  | *w* | 59 (34.5) | 46 (26.9) | 13 (7.6) | 0.027 * |
| **CCA subgroups** | *iCCA* | 72 (42.1) | 42 (24.6) | 30 (17.5) |  |
|  | *pCCA* | 56 (32.7) | 40 (23.4) | 16 (9.4) |  |
|  | *dCCA* | 43 (25.1) | 32 (18.7) | 11 (6.4) | 0.153 * |
| **Histology** | *ductal* | 146 (85.4) | 96 (56.1) | 50 (29.2) |  |
|  | *papillary* | 11 (6.4) | 7 (4.1) | 4 (2.3) |  |
|  | *mucinous* | 2 (1.2) | 1 (0.6) | 1 (0.6) |  |
|  | *intestinal* | 6 (3.5) | 4 (2.3) | 2 (1.2) |  |
|  | *other* | 6 (3.5) | 6 (3.5) | 0 (0.0) | 0.493 * |
| **UICC#** | *UICC 1* | 11 (6.4) | 5 (2.9) | 6 (3.5) |  |
|  | *UICC 2* | 58 (33.9) | 40 (23.4) | 18 (10.5) |  |
|  | *UICC 3* | 45 (26.3) | 36 (21.1) | 9 (5.3) |  |
|  | *UICC 4* | 16 (9.4) | 12 (7.0) | 4 (2.3) |  |
|  | *NA* | 41 (24.0) | 21 (12.3) | 20 (11.7) | 0.147 * |
| **pT** | *T1* | 21 (12.3) | 8 (4.7) | 13 (7.6) |  |
|  | *T2* | 92 (53.8) | 62 (36.3) | 30 (17.5) |  |
|  | *T3* | 43 (25.1) | 30 (17.5) | 13 (7.6) |  |
|  | *T4* | 15 (8.8) | 14 (8.2) | 1 (0.6) | 0.005 * |
| **pN** | *N0* | 55 (32.2) | 36 (21.1) | 19 (11.1) |  |
|  | *N1* | 71 (41.5) | 53 (31.0) | 18 (10.5) |  |
|  | *Nx* | 45 (26.3) | 25 (14.6) | 20 (11.7) | 0.325 * |
| **M** | *M0* | 155 (90.6) | 102 (59.6) | 53 (31.0) |  |
|  | *M1* | 16 (9.4) | 12 (7.0) | 4 (2.3) | 0.583 ** |
| **G** | *G1* | 8 (4.7) | 4 (2.3) | 4 (2.3) |  |
|  | *G2* | 121 (70.8) | 79 (46.2) | 42 (24.6) |  |
|  | *G3* | 42 (24.6) | 31 (18.1) | 11 (6.4) | 0.349 * |
| **L** | *L0* | 88 (51.5) | 55 (32.2) | 33 (19.3) |  |
|  | *L1* | 83 (48.5) | 59 (34.5) | 24 (14.0) | 0.259 * |
| **V** | *V0* | 125 (73.1) | 84 (49.1) | 41 (24.0) |  |
|  | *V1* | 46 (26.9) | 30 (17.5) | 16 (9.4) | 0.856 * |
| **R** | *R0* | 79 (46.2) | 47 (27.5) | 32 (18.7) |  |
|  | *R1* | 54 (31.6) | 38 (22.2) | 16 (9.4) |  |
|  | *R2* | 13 (7.6) | 10 (5.8) | 3 (1.8) |  |
|  | *Rx* | 25 (14.6) | 19 (11.1) | 6 (3.5) | 0.304 * |
| **Pn** | *Pn0* | 99 (57.9) | 59 (34.5) | 40 (23.4) |  |
|  | *Pn1* | 72 (42.1) | 55 (32.2) | 17 (9.9) | 0.023 * |

^§^ Median age: 63.6 years

* Fisher's exact test; not available data (NA).

^#^ Cases with pNx had no lymph nodes resected, therefore, UICC status could not be assessed.

**Supplemental Table 2:** *Surgical procedure and pre-existing conditions of patient cohort*

|  |  | Number (percent) | |
| --- | --- | --- | --- |
|  | Total | 171 (100.0) | |
| Type of surgery | Whipple | 31 (18.1) | |
|  | Hemihepatectomy | 80 (46.8) | |
|  | Partial hepatectomy | 16 (9.4) | |
|  | Bile duct resection | 16 (9.4) | |
|  | Liver transplantation | 2 (1.2) | |
|  | NA | 26 (15.2) | |
| Pre-existing hepatobiliary | Viral infection | 18 (10.5) | |
| conditions* | Liver chirrosis | 3 (1.8) | |
|  | Liver fibrosis | 2 (1.2) | |
|  | Steatohepatitis | 7 (4.1) | |
|  | Cholecystolithiasis | 21 (12.3) | |
|  | Choledocholithiasis | 4 (2.3) | |
|  | Cholecystitis | 8 (4.7) | |
|  | Hemochromatosis | 1 (0.6) | |
|  | Other | 3 (1.8) | |
|  | None diagnosed | 88 (51.5) | |
|  | NA | 24 (14.0) | |
| Autoimmune disease | Yes | 8 (4.7) | |
|  | None diagnosed | 145 (84.8) | |
|  | NA | 18 (10.5) | |
| NA: not available; * two conditions occurred in 8 patients each | | |  |


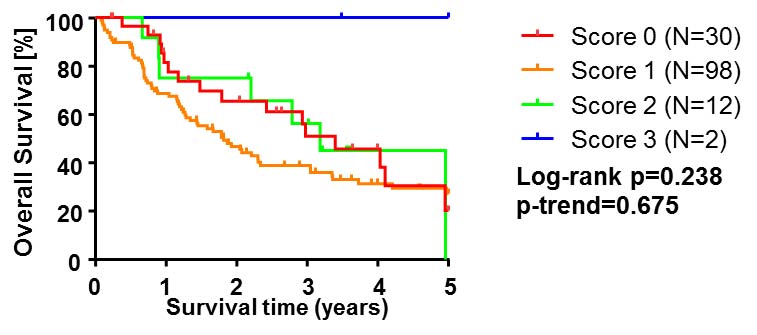


**Supplemental Figure 1:** *Overall survival probability in cholangiocarcinoma patients in correlation with CES2 immunoreactivity in tumor stroma.*

Kaplan-Meier curves show no significant overall survival differences of CCA patients in correlation with higher stromal CES2 immunoreactive scores. P-values were calculated by log-rank test or p-trend. Survival data were available for 142 of 171 (83%) CCA patients.


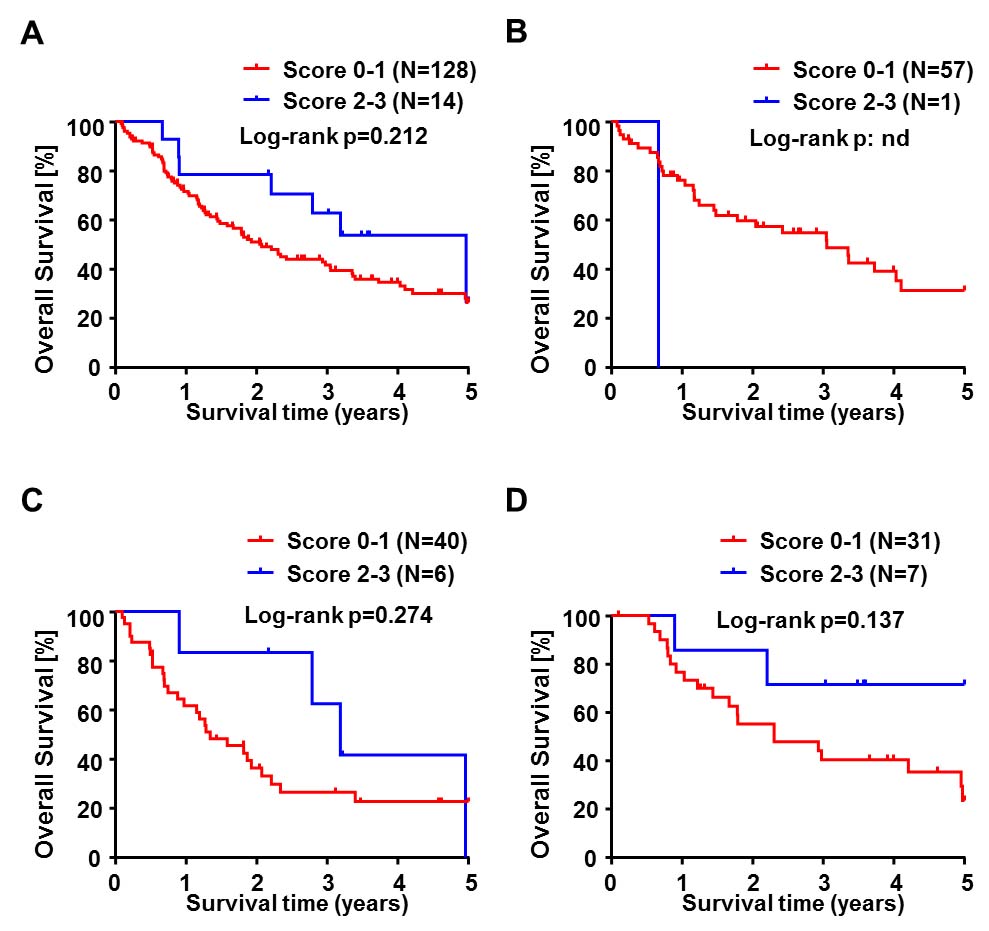


**Supplemental Figure 2:** *Overall survival probability in cholangiocarcinoma patients in correlation with CCA subtypes and CES2 immunoreactivity in tumor stroma.*

CES2 expression in tumor stroma of the CCA cohort (A), in iCCA (B), in pCCA (C), and in dCCA (D). P-values were calculated by log-rank test or p-trend. Survival data were available for 142 of 171 (83%) CCA patients.


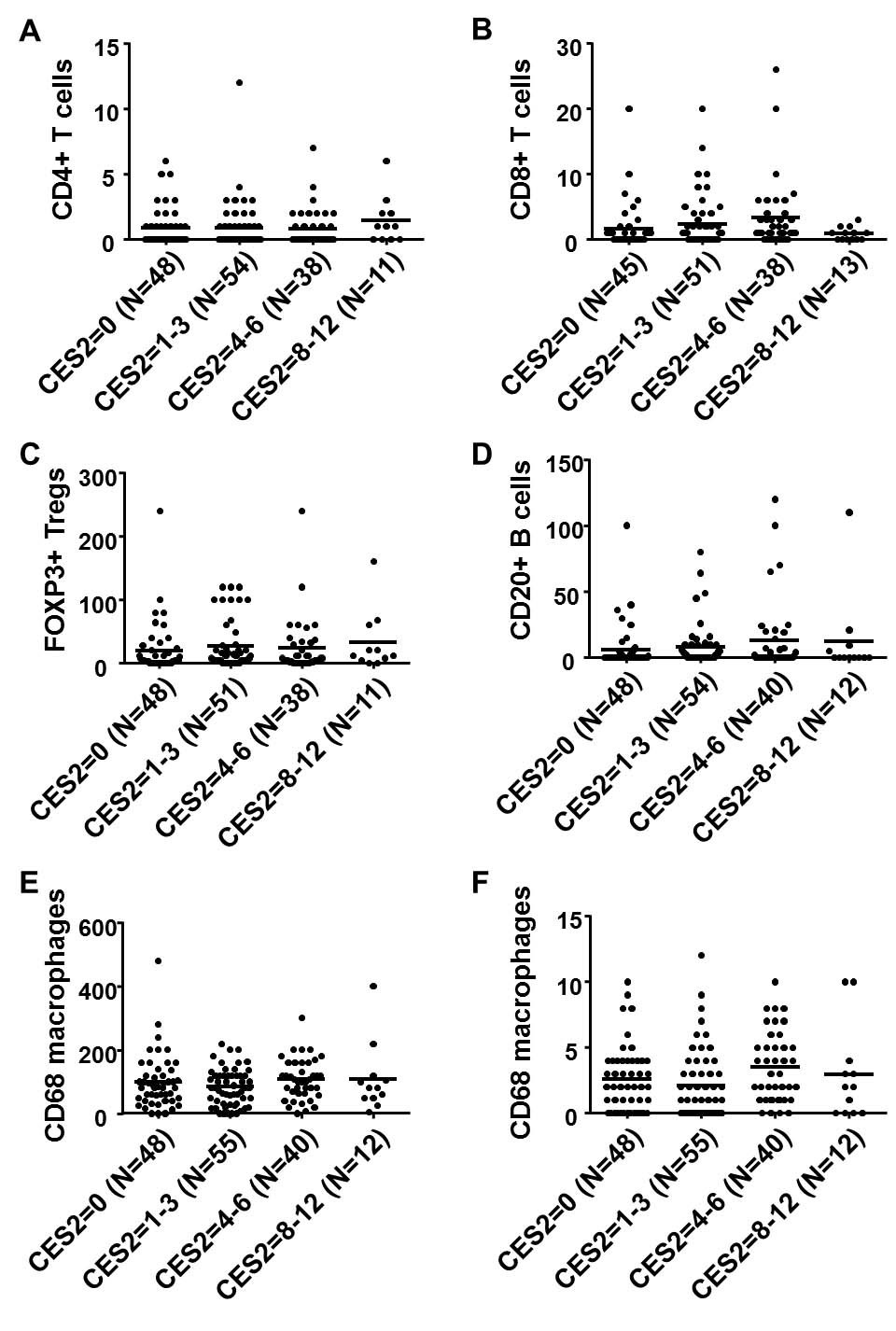


**Supplemental Figure 3:** *Immune cell infiltration in regard to CES2 expression.*

Analysis of quantity of CD4+ intraepithelial T cells in correlation with CES2 expression score in the CCA cohort (A), of CD8+ intraepithelial T cells (B), of total FOXP3+ regulatory T cells (C), of total CD20+ intraepithelial B cells (D), of total CD68+ macrophages (E), and of intraepithelial CD68+ macrophages (F) in the CCA cohort.


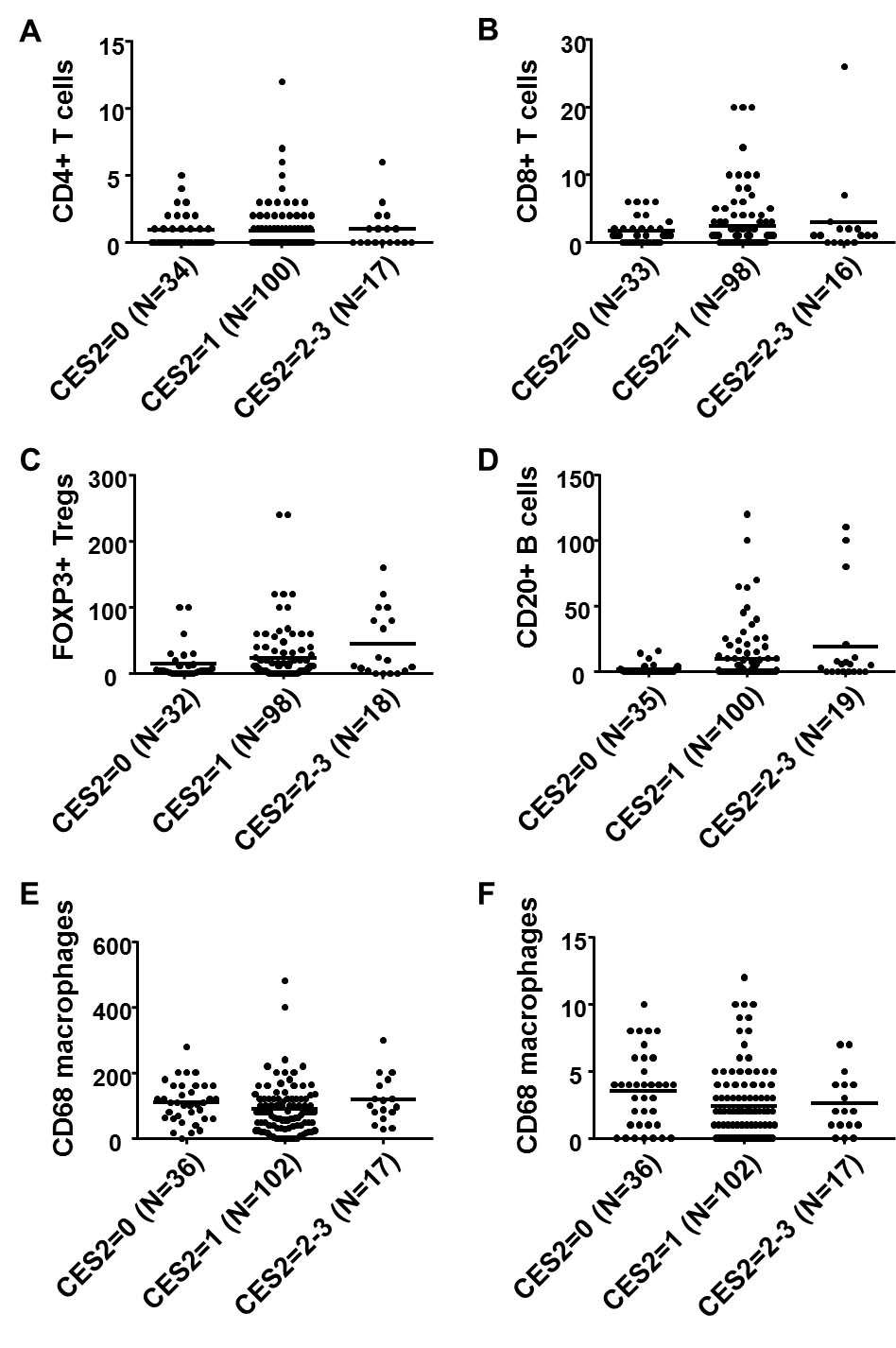


**Supplemental Figure 4:** *Immune cell infiltration in regard to stromal CES2 expression.*

Analysis of quantity of CD4+ intraepithelial T cells in correlation with stromal CES2 expression score in the CCA cohort (A), of CD8+ intraepithelial T cells (B), of total FOXP3+ regulatory T cells (C), of total CD20+ intraepithelial B cells (D), of total CD68+ macrophages (E), and of intraepithelial CD68+ macrophages (F) in the CCA cohort.


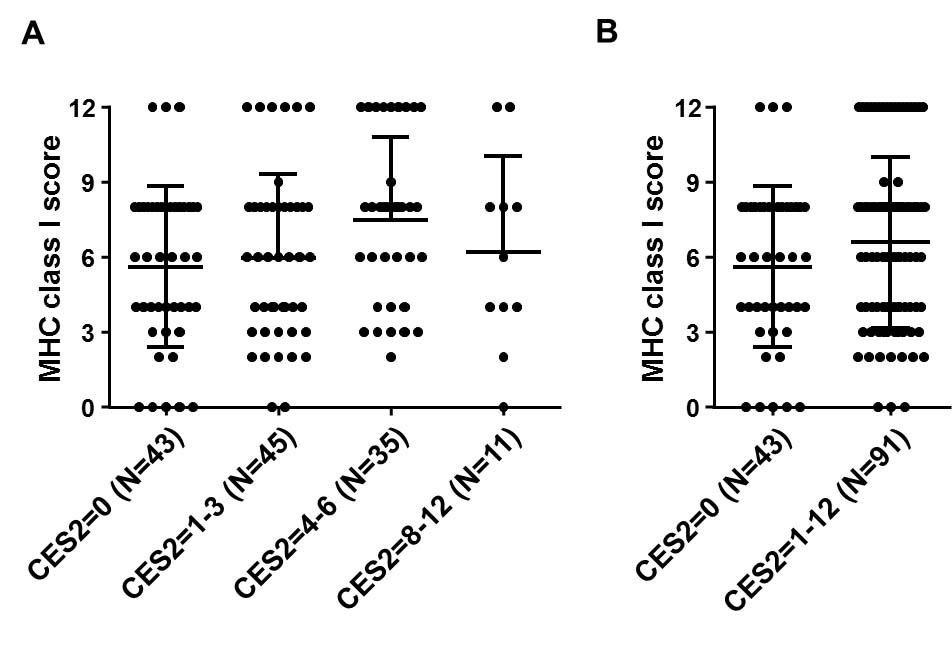


**Supplemental Figure 5:** *MHC class I expression in regard to CES2 expression in CCA.*

Analysis of MHC class I expression in tumor cells in correlation with tumoral CES2 expression score in the CCA cohort, displaying all CES2 expression subgroups (A), and dividing into negative and positive groups of CES2 expression (B).
